# Serum ^1^H nuclear magnetic resonance–based metabolomics of sole lesion development in Holstein cows

**DOI:** 10.3168/jds.2022-22681

**Published:** 2023-04

**Authors:** Matthew Barden, Marie M. Phelan, Robert Hyde, Alkiviadis Anagnostopoulos, Bethany E. Griffiths, Cherry Bedford, Martin Green, Androniki Psifidi, Georgios Banos, Georgios Oikonomou

**Affiliations:** 1 Department of Livestock and One Health, Institute of Infection, Veterinary and Ecological Sciences, University of Liverpool, Leahurst Campus, Liverpool, CH64 7TE, United Kingdom; 2 Institute of Systems, Molecular and Integrative Biology, University of Liverpool, Liverpool, L69 7ZB, United Kingdom; 3 High Field NMR Facility, Liverpool Shared Research Facilities University of Liverpool, Liverpool, L69 7ZB, United Kingdom; 4 School of Veterinary Medicine and Science, University of Nottingham, Sutton Bonington Campus, Leicestershire, LE12 5RD, United Kingdom; 5 Department of Clinical Science and Services, Royal Veterinary College, North Mymms, Hertfordshire, AL9 7TA, United Kingdom; 6 Animal & Veterinary Sciences, SRUC, Roslin Institute Building, Easter Bush, Midlothian, EH25 9RG, United Kingdom

**Keywords:** lameness, claw horn lesions, metabolomics, nuclear magnetic resonance (NMR) spectroscopy

## Abstract

Sole hemorrhage and sole ulcers, referred to as sole lesions, are important causes of lameness in dairy cattle. We aimed to compare the serum metabolome of dairy cows that developed sole lesions in early lactation with that of cows that remained unaffected. We prospectively enrolled a cohort of 1,169 Holstein dairy cows from a single dairy herd and assessed animals at 4 time points: before calving, immediately after calving, early lactation, and late lactation. Sole lesions were recorded by veterinary surgeons at each time point, and serum samples were collected at the first 3 time points. Cases were defined by the presence of sole lesions in early lactation and further subdivided by whether sole lesions had been previously recorded; unaffected controls were randomly selected to match cases. Serum samples from a case-control subset of 228 animals were analyzed with proton nuclear magnetic resonance spectroscopy. Spectral signals, corresponding to 34 provisionally annotated metabolites and 51 unlabeled metabolites, were analyzed in subsets relating to time point, parity cohort, and sole lesion outcome. We used 3 analytic methods (partial least squares discriminant analysis, least absolute shrinkage and selection operator regression, and random forest) to determine the predictive capacity of the serum metabolome and identify informative metabolites. We applied bootstrapped selection stability, triangulation, and permutation to support the inference of variable selection. The average balanced accuracy of class prediction ranged from 50 to 62% depending on the subset. Across all 17 subsets, 20 variables had a high probability of being informative; those with the strongest evidence of being associated with sole lesions corresponded to phenylalanine and 4 unlabeled metabolites. We conclude that the serum metabolome, as characterized by proton nuclear magnetic resonance spectroscopy, does not appear able to predict sole lesion presence or future development of lesions. A small number of metabolites may be associated with sole lesions although, given the poor prediction accuracies, these metabolites are likely to explain only a small proportion of the differences between affected and unaffected animals. Future metabolomic studies may reveal underlying metabolic mechanisms of sole lesion etiopathogenesis in dairy cows; however, the experimental design and analysis need to effectively control for interanimal and extraneous sources of spectral variation.

## INTRODUCTION

Sole hemorrhage (**SH**) and sole ulcers (**SU**), referred to as sole lesions, are 2 of the most prevalent foot lesions in dairy cattle (Murray et al., 1996; Capion et al., 2008; Cramer et al., 2008). Sole lesions are thought to arise from contusions in the corium following a failure of the suspensory and supportive apparatus of the distal phalanx (Ossent and Lischer, 1998; Lischer et al., 2002; Bicalho and Oikonomou, 2013, Breiman, 2001). Tissue damage in the corium can manifest as blood staining of the sole horn or impairment of horn production, presenting as SH or SU, respectively (Hoblet and Weiss, 2001; Lischer and Ossent, 2002; Shearer and van Amstel, 2017).

Sole lesions occur more frequently in hindlimbs and the incidence peaks around 3 to 4 mo after calving (Leach et al., 1997; Offer et al., 2000; Barker et al., 2009). As there is an assumed lag of approximately 2 mo between instigating pathology in the corium and the detection of visible sole lesions (Hoblet and Weiss, 2001), much of the research concerning the etiopathogenesis of sole lesions has focused on the transition period and early lactation. This represents a time of significant nutritional, metabolic, and management changes in dairy cattle (Drackley, 1999; Overton and Waldron, 2004; Cook and Nordlund, 2009). Therefore, although numerous possible metabolic mechanisms could have a role in the development of sole lesions, the interconnecting relationships between causal factors and predisposing or exacerbating risks are complex, with many of these elements still lacking a strong evidence base (Solano et al., 2015; Newsome et al., 2016).

Measurement of metabolic markers in the blood has long been recognized as a valuable tool to monitor periparturient and early lactation dairy cows (Payne et al., 1970; Ingraham and Kappel, 1988). More recently, the field of metabolomics has been established using high-throughput platforms to detect large numbers of low-molecular-weight metabolites within a biological sample (Wishart, 2008). The primary technologies used in metabolomics are chromatographic separation coupled with mass spectrometry or proton nuclear magnetic resonance (**^1^H NMR**) spectroscopy (Emwas et al., 2019).

Metabolomic techniques have only recently been directed toward studying lameness in dairy cows. To date, most research in this area comes from a single case-control study of 6 lame cows and 20 nonlame controls that generated multiple ^1^H NMR- and MS-based metabolic analyses of serum, milk, and urine (Dervishi et al., 2020; Eckel et al., 2020; Zhang et al., 2020a,b; Zwierzchowski et al., 2020). These studies highlighted large numbers of metabolites that may be associated with lameness, both several weeks before and after the onset of clinical signs. Results also implied that metabolic markers could be used to discriminate between lame and nonlame cows with almost perfect accuracy in all evaluated biofluids. However, as the cause of lameness was not described in these studies, it is unclear whether these results directly relate to sole lesions. Nevertheless, there appears to be potential for metabolomic approaches to highlight metabolic pathways implicated in lameness, which could make a valuable contribution toward understanding the etiopathogenesis of sole lesions.

This study aimed to compare the serum metabolome of dairy cows that developed SH and SU (sole lesions) in early lactation with the metabolome of cows that remained unaffected. We aimed to (1) determine whether the serum metabolome could predict the presence, or future development, of SH and SU; and (2) identify any informative metabolites related to these lesions. Within the overarching aims, specific objectives also included (a) assessment of the serum metabolome before the development of sole lesions, particularly around parturition; (b) assessment of the serum metabolome in animals with a concurrent sole lesion; and (c) specific evaluation of first-parity animals, which are less likely to have had previous cases of severe sole lesions.

## MATERIALS AND METHODS

This study was conducted following ethical approval by the University of Liverpool Research Ethics Committee (VREC269a, VREC466ab), and procedures regulated by the Animals (Scientific Procedures) Act were conducted under a United Kingdom Home Office License (P191F589B).

### Study Overview

We designed a 2-stage observational study to evaluate the serum metabolome of dairy cattle that developed sole lesions in early lactation. The first stage followed a prospective cohort approach to record foot lesions and collect serum samples at repeated time points during a production cycle. In the second stage, a case-control sampling strategy was used to select animals, primarily based on sole lesions recorded in early lactation, from which to analyze serum samples with ^1^H NMR spectroscopy. Statistical analysis aimed to assess whether the serum metabolome could differentiate between cases and controls, and to highlight any metabolites that were influential in this discrimination.

### Stage 1: Cohort Study

#### Herd Description.

Data collection was conducted on a single dairy herd in the northwest United Kingdom, which was chosen for convenience and practical reasons, including the feasibility of frequent visits and assessments, herd size (>1,000 cows), and the availability of a suitable environment on the farm in which to process serum samples. Animals were housed year round in freestall barns with grooved concrete passageways and cubicles deep bedded with sand. Cows were milked 3 times daily, and the average 305-d milk yield was approximately 11,000 kg at 3.3% protein and 3.8% fat. All animals were fed ad libitum with a TMR formulated for each management group; no additional feed was provided during milking. In this herd, cows were routinely foot-trimmed before drying off and in early lactation. Foot-trimming was conducted by experienced farm staff using a grinder and hoof knives. Farm staff had been trained to use a modified version of the 5-step Dutch foot-trimming method (Toussaint Raven et al., 1985), which included wider and deeper modeling of the lateral claw on hind feet than the traditional method. The timing of early lactation foot-trimming depended on parity: primiparous cows were foot-trimmed 60 to 70 d postpartum and multiparous cows were foot-trimmed 90 to 100 d postpartum. Cows were footbathed twice daily with a 2% formalin solution.

#### Enrollment and Study Design.

All animals that were expected to calve between May and December 2019 were prospectively enrolled with no additional inclusion or exclusion criteria applied. A total of 1,169 animals were enrolled. Data were collected by qualified veterinary surgeons during twice-weekly visits from April 2019 to July 2020 (with a break from March to June 2020 due to COVID-19 restrictions). Animals were assessed at 4 time points: −90 to −21 d relative to parturition (**T1-Precalving**), 1 to 10 d after parturition (**T2-Calving**), 50 to 120 d in lactation (**T3-Early**), and >170 d in lactation (**T4-Late**). The timing of T3-Early corresponded to the scheduled foot-trimming protocol on the farm; therefore, this time point was slightly earlier for first-lactation animals than for older animals. Serum samples were collected at the first 3 time points (T1-Precalving, T2-Calving, and T3-Early). To maximize the number of cases available for the subsequent case-control sampling, we aimed to enroll as many animals as possible during this stage; all eligible animals were enrolled until the final assessments (T4-Late) began, at which point further enrollments stopped as data collection at 4 time points simultaneously was not feasible.

#### Data Collection Procedures.

**Figure 1:**
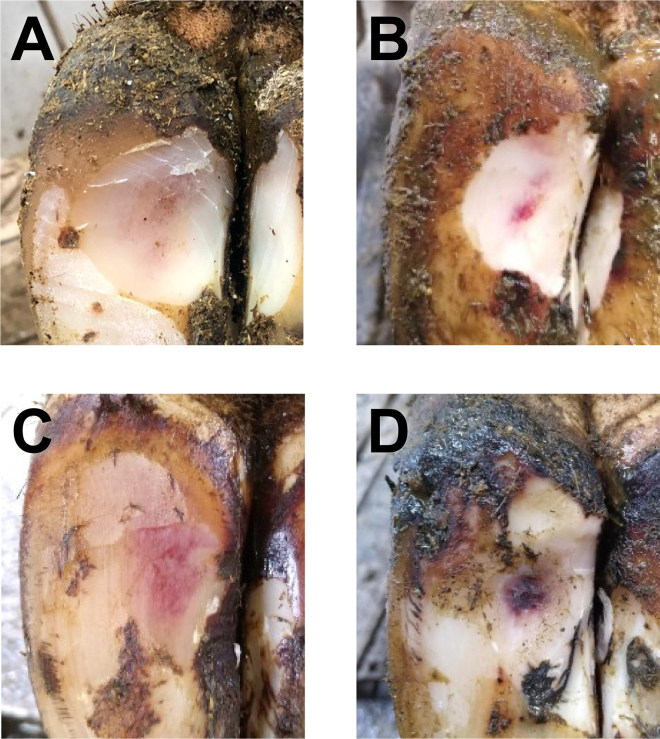
Examples of sole hemorrhage (SH) severity grading. Mild SH: diffuse discoloration of sole (A) or a light pink lesion <2 cm diameter (B); severe SH: light pink lesion ≥2 cm diameter (C) or dark pink/purple lesion of any size (D).

Data collection was always conducted at the same time of day in nulliparous animals and immediately after milking in parous animals; therefore, sampling times relative to feed access were consistent within contemporary groups. At each assessment, animals were restrained in a foot-trimming crush. Body condition score was recorded using a 1-to-5 scale with quarter-point intervals (Ferguson et al., 1994). Foot lesions were recorded either during routine foot-trimming (T1-Precalving and T3-Early) or after lightly trimming the sole horn to visualize lesions (T2-Calving and T4-Late). On each claw, all foot lesions were recorded using case definitions as described in the International Committee for Animal Recording (ICAR) *Claw Health Atlas* (Egger-Danner et al., 2020). All foot lesions were examined and recorded by qualified veterinary surgeons; over 95% were by a single researcher and the remainder by 3 other researchers. Sole hemorrhage was graded as either mild (light pink lesion <2 cm diameter or diffuse discoloration of sole) or severe (light pink lesion ≥2 cm diameter or dark pink/purple lesion of any size; Figure 1). Sole ulcers were recorded as present or absent. Foot lesion recording was the same at all time points except for T2-Calving, during which only hind feet were assessed to reduce the handling time of recently calved cows. Painful sole lesions were treated according to herd protocols, which always included therapeutic trimming and the application of a hoof block to the unaffected claw if there was exposure of the corium, a pain response was elicited following pressure on the lesion, or the animal had impaired mobility attributable to the lesion. Bandages were not applied to sole lesions.

At the first 3 time points (T1-Precalving, T2-Calving, and T3-Early), blood samples were collected from the ventral coccygeal vein into plastic Vacutainers coated with silica (Becton Dickinson Ltd.). Samples were mixed, allowed to clot at room temperature, and then centrifuged at 1,300 × *g* for 20 min to separate the serum. Serum aliquots were either transported on ice directly to −80°C storage or placed into a −20°C freezer on the farm for up to 8 h before being transferred to storage at −80°C. The times between sampling and centrifugation and between centrifugation and storage at −80°C were recorded.

The maximum daily milk yield during the first 100 d after calving was obtained from farm records for each animal.

### Stage 2: Case-Control Study

**Figure 2:**
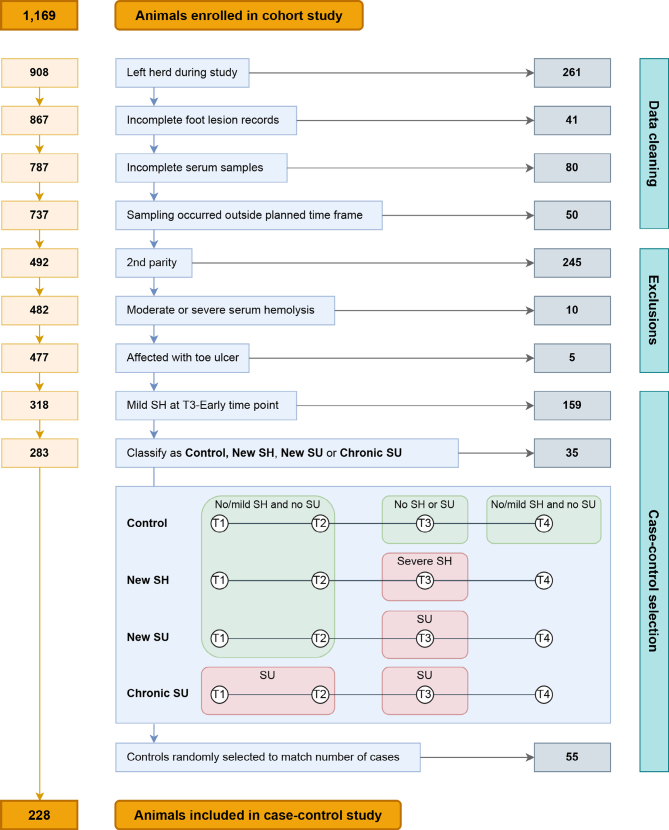
Data handling workflow to create the final case-control study population used in the analysis. The running total of animals in the data set is shown in the orange box on the left, the reason for animal exclusion is shown in the blue box in the middle, and the number of animals excluded at each step is shown in the gray box on the right. SH = sole hemorrhage; SU = sole ulcer; T1 = precalving; T2 = calving; T3 = early lactation; and T4 = late lactation.

Resources were available to analyze up to 600 serum samples using ^1^H NMR spectroscopy; therefore, a case-control approach was used to select samples for further analysis from the data collected during stage 1 of this study. Data handling and case-control selection are summarized in Figure 2.

#### Data Cleaning.

The data set was filtered to exclude animals with missing data. Missing data occurred if the animal left the herd during the study period, foot lesion records were incomplete from an assessment (minimum requirement of lesion records from both hindfeet), or a serum sample was missing from one of the first 3 time points. Animals were also excluded if any data collection had occurred outside of the planned sampling times previously described. After data cleaning, a total of 727 animals with complete lesion records and serum samples had been sampled according to the planned timeline.

#### Exclusions.

We defined a cohort of first-parity animals to specifically evaluate animals that were unlikely to have been affected by previous SU or other noninfectious foot lesions (Vermunt and Greenough, 1996; Randall et al., 2016). A low incidence of both SH and SU was recorded in second-parity animals, so these animals were excluded from subsequent analysis. Animals that were third parity or greater were grouped into a separate cohort.

Serum samples from T2-Calving and T3-Early were analyzed in both parity cohorts (first parity and ≥ third parity), and samples from T1-Precalving were analyzed in the first-parity cohort only. At T1-Precalving, nulliparous heifers were considered likely to be relatively homogeneous, unlike parous animals, and therefore presented the best opportunity to evaluate possible differences in the metabolome before parturition.

Severe serum hemolysis is associated with changes in the metabolome (Denihan et al., 2015). Before the final selection of cases and controls, serum samples were assessed for serum hemolysis using a 0 to 4 visual grading system (Akinyemi et al., 2018). A conservative threshold was applied so that animals with samples that had hemolysis of grades 3 or 4 were excluded from further analysis.

Toe ulcers may develop from similar pathophysiological processes to sole ulcers, but can also occur due to over-wear or excessive trimming (Sanders et al., 2009; Kofler, 2017). Only a small number of cows were affected with toe ulcers (n = 5), so these were excluded to avoid potentially confounding effects.

#### Case and Control Classification.

Cases and controls were defined from 477 eligible animals following data cleaning and exclusions. Our principal case definition was “severe sole lesions in early lactation,” but to address the research objectives, we subclassified cases according to more specific case definitions (Figure 2):

1. New SH: severe SH present at T3-Early; unaffected with severe SH or SU at T1-Precalving and T2-Calving.
2. New SU: SU present at T3-Early; unaffected with severe SH or SU at T1-Precalving and T2-Calving.
3. Chronic SU: affected with SU at either T1-Precalving or T2-Calving, in addition to T3-Early. This case definition was included to differentiate between animals with and without SU before T3-Early, but allowed all animals with SU at T3-Early (i.e., New SU and Chronic SU) to be assessed.

Control animals were defined as those without any grade of SH or SU at T3-Early, in addition to being unaffected with severe SH or SU at T1-Precalving, T2-Calving, and T4-Late. In other words, at T3-Early, cases had either a new occurrence of severe SH or any occurrence of SU, and controls had no sole lesions at all. Once cases had been defined, the same number of controls were randomly selected from eligible animals within each parity cohort. A total of 114 animals were classified as cases and an equal number of controls were randomly selected from eligible animals.

### ^1^H NMR Spectroscopy

#### NMR Spectra Acquisition and Processing.

Full details of NMR spectra acquisition and processing are provided in the Supplemental Materials (https://data.mendeley.com/datasets/dgrb5z38kb/1; Barden, 2023). Briefly, following sample preparation, spectra were acquired using a Bruker Ascend 700 MHz. One-dimensional ^1^H NMR spectra were recorded using a Carr-Purcell Meiboom-Gill (**CPMG**) pulse sequence (cpmgpr1d, Bruker). Quality control assessments included the measurement of the half-height linewidth of glucose at 5.24 ppm, as well as a visual determination of acceptable water suppression, baseline stability, and signal-to-noise ratio. If a sample failed quality control, then the spectrum acquisition process was repeated up to 5 times per sample, after which samples were excluded from further analysis. Fourteen samples repeatedly failed quality control procedures and were excluded from further analysis. Thirteen of these samples were from the T3-Early assessment time point, and 1 was from T2-Calving; 7 of these samples were from first-parity animals, and the other 7 were from ≥ third-parity animals. The final data set used for statistical analysis comprised 567 samples from 228 animals.

#### NMR Spectra Binning and Annotation.

The signal intensity of each peak is directly proportional to the concentration of each metabolite, as well as the number of hydrogen ions in that molecule (Emwas et al., 2019). No reference standards were used to calculate the absolute concentrations of each metabolite; therefore, signal intensities represent the relative concentrations (Cullen et al., 2013).

Spectra from all samples were overlaid using the tameNMR toolkit on an in-house Galaxy server (Afgan et al., 2018; tameNMR, 2021), which allowed visualization of all small and consistent signals. Spectral signals were provisionally annotated using Chenomx software (version 8.2; Chenomx Inc.). Annotations were based on chemical shift and multiplicity using the Chenomx reference library of mammalian metabolites and cross-referenced with the Bovine Metabolome Database (Foroutan et al., 2020).

Bin boundaries were manually specified to capture annotated peaks; if metabolite annotation was not possible, then boundaries were selected to include individual peaks, distinct multiplets, or regions of consistent deviation from the baseline in all overlaid spectra. The peaks within each bin were integrated to calculate the relative intensity of each metabolite. Data were imported into R for subsequent analysis (R Core Team, 2021). Any negative values (due to noise at the baseline) were replaced with one-fifth of the minimum positive value for each bin. Data from each sample were normalized by the total spectral intensity to reduce technical variance between samples. A total of 211 bins were defined, which contained all spectral peaks unaffected by residual water signal; 118 bins were provisionally annotated to 34 different metabolites.

#### Bin Selection.

Twenty-four labeled metabolites were represented by multiple bins in the spectrum. For each of these metabolites, a single bin was manually selected that was highly correlated with other bins of the same metabolite label, and therefore considered to be representative of that metabolite (Grosman, 2019). Representative peaks were also selected based on the confidence of annotation and visually appraised to select a bin with minimal visible overlap from neighboring signals.

Ninety-three bins could not be annotated with a candidate metabolite. Unlabeled bins were excluded if they were strongly correlated with a labeled bin (Pearson correlation coefficient ≥0.9). The remaining unlabeled bins were assessed for distinct clusters based on Euclidean distances using complete-linkage hierarchical clustering. The number of clusters was selected so that bins in different clusters had a Pearson correlation coefficient <0.9; a single bin within each cluster was selected based on the clarity of peak isolation, as before.

The result of bin selection was to define the spectrum by a total of 85 bins (Supplemental Tables S1 and S2, https://data.mendeley.com/datasets/dgrb5z38kb/1; Barden, 2023). All 34 annotated metabolites were represented by a single bin, and the remaining 51 unlabeled bins were not strongly correlated with labeled or other unlabeled bins. The relative intensities calculated for the signals in each selected bin were used for subsequent statistical analysis, referred to hereafter as (explanatory) variables.

### Statistical Analysis

Statistical analysis was conducted in R using the *tidyverse* and *tidymodels* packages (Wickham et al., 2019; Kuhn and Wickham, 2020) in addition to those specified. Full details of our approach to statistical analysis are provided in the Supplemental Materials (https://data.mendeley.com/datasets/dgrb5z38kb/1; Barden, 2023), and key aspects are described below.

#### Preliminary Analysis.

Principal component analysis (**PCA**) was conducted with the *GGally* package (Schloerke et al., 2018). The first 5 principal components, of centered and autoscaled data (van den Berg et al., 2006), were assessed for clustering or correlation due to technical or biological confounders. Technical confounders assessed were the timing of sampling relative to parturition, the time between sampling and centrifugation, the time between centrifugation and storage at −80°C, and the degree of serum hemolysis. Biological confounders assessed were BCS, maximum daily milk yield, and the presence of other foot lesions.

#### General Approach to Statistical Analysis.

To address our research objectives, we split the data set to evaluate the relationship between the serum metabolome and sole lesion development in subsets of comparable animals. We defined 4 clinically relevant outcomes from the case definitions of new SH, new SU, and chronic SU: (1) new SH, (2) new SU, (3) either new SH or new SU (new SH/SU), and (4) either new SU or chronic SU (all SU). We created separate subsets for each of these 4 outcomes and further divided these by assessment time point (T1-Precalving, T2-Calving, T3-Early) and parity cohort (first parity and ≥ third parity). In all instances, the comparison (control) group was cows that were not affected with severe SH or SU at any time point. Consequently, we conducted statistical analysis in 17 subsets in which the total number of animals ranged from 58 to 211.

#### Univariable Analysis.

The distribution of the relative intensity of each metabolite in each of the predefined subsets was visualized with density plots. Differences between case and control groups were assessed with a 2-tailed, unpaired Wilcoxon signed-rank test, as the distribution was non-Gaussian in many instances.

#### Multivariable Analysis.

The 3 statistical methods used for multivariable analysis were partial least squares discriminant analysis (**PLSDA**), least absolute shrinkage and selection operator (**Lasso**) regression, and random forest classification; these were applied separately to each predefined subset. In all cases, model predictive performance was assessed using balanced accuracy, calculated as the average accuracy for each class. Model hyperparameters were tuned to optimize balanced accuracy via 5-fold cross-validation repeated 20 times; the balanced accuracy of class prediction was assessed from the out-of-fold prediction results. The same 3 methods were also used to highlight explanatory variables with an informative association with the outcome in each subset.

The PLSDA models were fit on centered and autoscaled data (van den Berg et al., 2006) using the *mixOmics* package (Rohart et al., 2017). Variable importance in projection (VIP) was calculated and a variable was considered to be selected if the VIP score was >1. Logistic regression models with the Lasso penalty were fit using the *glmnet* package (Friedman et al., 2010) on centered and autoscaled data (van den Berg et al., 2006). A variable was considered to be selected if there was a nonzero coefficient in the final model. Random forests were fit using the *ranger* package (Wright and Ziegler, 2017). The Boruta algorithm fits a random forest and selects variables that have significantly higher importance scores than permuted variables (*P* < 0.01). The Boruta algorithm was implemented using the *Boruta* package (Kursa and Rudnicki, 2010).

#### Observed Stability.

The robustness of selected variables to small perturbations in the data was assessed by bootstrapping the data (random sampling of the data with replacement) and repeating the variable selection steps on each bootstrapped resample. The proportion of times a variable is selected, termed “stability,” reflects the confidence in a selected variable being truly informative (Austin and Tu, 2004; Meinshausen and Bühlmann, 2010; Sauerbrei et al., 2015). We calculated the variable selection stability from 200 bootstrapped resamples of each subset using each of the 3 variable selection methods (PLSDA, Lasso regression, and Boruta). Triangulation of results can further strengthen inference (Lawlor et al., 2016). We took the average stability from the 3 variable selection methods to calculate a single, combined stability for each variable (Lima et al., 2021), referred to as the “observed stability.”

#### Baseline Stability.

In each subset, we calculated stability thresholds with permuted data to formalize the interpretation of the observed stability of each variable (Hyde et al., 2022). Following the approach described by Hyde et al. (2022), the outcome variable was permuted to create a data set in which any existing association between explanatory variables and the outcome had been removed. This was repeated 10 times to produce independent data sets with randomized class labels. In each of these permuted data sets, the previous steps of bootstrapping and variable selection were repeated using 20 bootstrapped resamples. As before, the variable selection stability in the bootstrapped resamples was calculated for each variable and then averaged over the 3 variable selection methods to determine the “baseline stability.”

In each permuted data set, the 99th and 100th percentiles of the baseline stabilities were taken from the distribution of baseline stabilities from all individual variables and then averaged over the 10 permuted data sets to define 2 thresholds, **T_99_** and **T_100_**, respectively. Therefore, on average, 1% of variables in permuted data sets had a baseline stability that exceeded the T_99_ threshold, analogous to an expected false-positive rate of 1%; similarly, the T_100_ threshold translates to an expected false-positive rate of 0%.

## RESULTS

A total of 1,169 animals were prospectively enrolled to record foot lesions and collect serum samples, with data from 737 animals retained following data cleaning (Figure 2). In the data set of 737 animals, 99.9% (736/737), 99.3% (732/737), and 96.6% (712/737) of animals had lesion records from all 4 feet at T1-Precalving, T3-Early, and T4-Late, respectively; all animals had lesion records from both hind feet at T2-Calving.

Details of foot lesion frequency at each assessment time point are provided in Table 1. At T3-Early, 5% (12/249) of primiparous animals and 11% (27/243) of ≥ third-parity animals had SU; only 1% (2/245) of second-parity animals had SU at this time point. Not including those animals that also had SU, 26% (65/249) of primiparous animals and 19% (46/243) of ≥ third-parity animals had severe SH at T3-Early; only 9% (21/245) of second-parity animals had severe SH at T3-Early.

**Table 1:** Proportion (frequency) of animals affected by sole hemorrhage (SH) and sole ulcers (SU) at each time point (T1–T4) in the study population following data cleaning (n = 737); animals were defined according to the most severe sole lesion on all claws

Time point	No sole lesion	Mild SH	Severe SH	SU
First parity				
T1-Precalving	0.74 (184)	0.24 (59)	0.02 (5)	<0.01 (1)
T2-Calving	0.74 (185)	0.20 (51)	0.05 (12)	<0.01 (1)
T3-Early	0.37 (92)	0.32 (80)	0.26 (65)	0.05 (12)
T4-Late	0.61 (152)	0.33 (83)	0.04 (11)	0.01 (3)
Second parity				
T1-Precalving	0.90 (221)	0.09 (22)	<0.01 (1)	<0.01 (1)
T2-Calving	0.87 (214)	0.11 (27)	0.02 (4)	0.00 (0)
T3-Early	0.62 (151)	0.29 (71)	0.09 (21)	0.01 (2)
T4-Late	0.61 (149)	0.31 (77)	0.05 (13)	0.02 (6)
≥Third parity				
T1-Precalving	0.63 (153)	0.23 (57)	0.06 (15)	0.07 (18)
T2-Calving	0.69 (167)	0.24 (58)	0.03 (7)	0.05 (11)
T3-Early	0.37 (91)	0.33 (79)	0.19 (46)	0.11 (27)
T4-Late	0.41 (100)	0.35 (84)	0.14 (34)	0.10 (25)

A total of 228 animals were classified as either cases or controls. In the first-parity cohort, the mean (standard deviation) maximum daily milk yield was 36.9 (5.0) kg and 38.3 (4.8) kg in cases and controls, respectively; in the ≥ third-parity cohort, the mean (standard deviation) maximum daily milk yield was 54.5 (6.4) kg and 54.7 (6.4) kg, respectively. Details of other potentially confounding factors in cases and controls are provided in Table 2, Table 3.

**Table 2:** Details of potential technical confounders in the case and control groups

Time point	N	Mean (SD) assessment timing relative to parturition (d)	Mean (SD) time (min) between		Proportion (frequency) of samples by hemolysis grade (0–4)		
			Sampling and serum separation	Serum separation and −80°C storage	Grade 0	Grade 1	Grade 2
First parity							
T1-Precalving							
Control	64	−52.6 (12.7)	119.8 (28.5)	405.1 (73.2)	0.70 (45)	0.25 (16)	0.05 (3)
Case	61	−53.3 (13.9)	120.1 (20)	405.6 (61.3)	0.61 (37)	0.31 (19)	0.08 (5)
T2-Calving							
Control	64	4.5 (2.0)	107.8 (64.8)	149.8 (81.6)	0.73 (47)	0.17 (11)	0.09 (6)
Case	61	5.8 (2.3)	92.7 (48.7)	171.9 (83.6)	0.70 (43)	0.18 (11)	0.11 (7)
T3-Early							
Control	60	67.0 (4.2)	49.5 (18.9)	143.9 (81.1)	0.60 (36)	0.35 (21)	0.05 (3)
Case	58	67.1 (7.9)	52.0 (24.6)	133.4 (68.8)	0.66 (38)	0.28 (16)	0.07 (4)
≥Third parity							
T2-Calving							
Control	49	5.4 (2.2)	72.6 (38.6)	192.5 (86.6)	0.78 (38)	0.16 (8)	0.06 (3)
Case	52	5.8 (2.4)	71.5 (25.8)	194.4 (81.1)	0.75 (39)	0.17 (9)	0.08 (4)
T3-Early							
Control	48	95.7 (6.6)	74.3 (32.2)	185.8 (83.5)	0.79 (38)	0.12 (6)	0.08 (4)
Case	50	95.0 (4.5)	83.7 (26.2)	190.5 (83.2)	0.82 (41)	0.12 (6)	0.06 (3)

**Table 3:** Details of potential biological confounders in the case and control groups; results are displayed as proportion affected (frequency) unless otherwise stated

Time point	Median (IQR^1^) BCS (1–5)	White line lesion	Thin sole	Double sole	Digital dermatitis	Heel horn erosion
First parity						
T1-Precalving						
Control	4 (4–4.25)	0.03 (2)	0.02 (1)	0.05 (3)	0.52 (33)	0.02 (1)
Case	4 (4–4.25)	0.07 (4)	0.03 (2)	0.05 (3)	0.51 (31)	0.02 (1)
T2-Calving						
Control	3.5 (3.5–3.75)	0.03 (2)	0.00 (0)	0.05 (3)	0.31 (20)	0.08 (5)
Case	3.5 (3.5–3.75)	0.03 (2)	0.00 (0)	0.03 (2)	0.26 (16)	0.02 (1)
T3-Early						
Control	3.25 (3–3.25)	0.03 (2)	0.12 (7)	0.13 (8)	0.18 (11)	0.12 (7)
Case	3.25 (3–3.5)	0.03 (2)	0.07 (4)	0.05 (3)	0.14 (8)	0.12 (7)
≥Third parity						
T2-Calving						
Control	3.5 (3.25–3.5)	0.02 (1)	0.00 (0)	0.04 (2)	0.18 (9)	0.08 (4)
Case	3.25 (3–3.5)	0.10 (5)	0.04 (2)	0.04 (2)	0.19 (10)	0.17 (9)
T3-Early						
Control	3.25 (3–3.25)	0.08 (4)	0.08 (4)	0.29 (14)	0.19 (9)	0.21 (10)
Case	3 (2.75–3.25)	0.14 (7)	0.10 (5)	0.32 (16)	0.20 (10)	0.34 (17)

1 Interquartile range.

**Figure 3:**
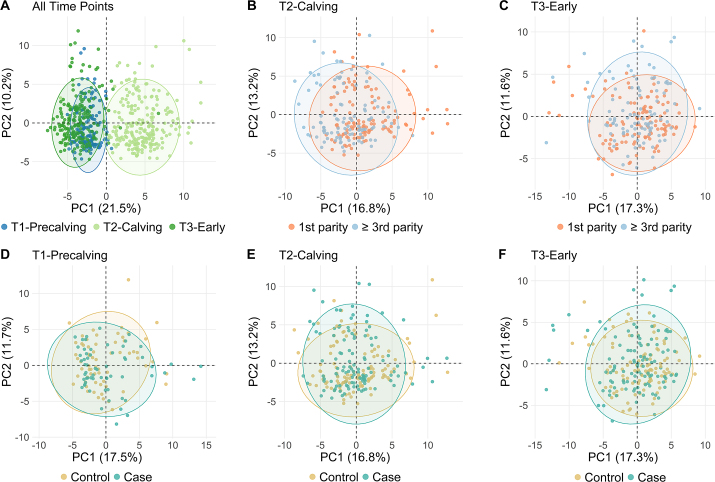
Unsupervised principal component analysis scores of the first 2 principal components (PC) for all time points together (A) and for separate time points (B–F). Points are colored by time point (A), parity cohort (B and C), and by case/control classification (D–F). Ellipses represent the 95% confidence level for a multivariate t-distribution.

Serum samples from the first-parity cohort at T1-Precalving, T2-Calving, and T3-Early, and from the ≥ third-parity cohort at T2-Calving and T3-Early were analyzed using ^1^H NMR spectroscopy. Plots of PCA scores for all time points combined and for each time point separately are shown in Figure 3. Potential technical and biological confounders (Table 2, Table 3) were assessed with PCA, and no clustering or correlation attributable to these confounders was evident in the first 5 principal components.

The full data set was split by time point, parity, and outcome definition to create 17 subsets that related to the specific comparisons of interests (Table 4). The number of animals ranged from 58 to 211 in each subset. Univariable analyses of explanatory variables in each subset were conducted with a Wilcoxon signed-rank test to compare cases and controls (Supplemental Table S3, https://data.mendeley.com/datasets/dgrb5z38kb/1; Barden, 2023). In multivariable analyses, the balanced accuracy of class prediction was low in all subsets regardless of the statistical method; the average balanced accuracy of the 3 methods in each subset ranged from 50 to 62% (Table 4).

**Table 4:** Balanced accuracy of class prediction using partial least squares discriminant analysis (PLSDA), Lasso regression (Lasso), random forests (RF), and the average of all 3 methods (Combined)^1^

Time point	Control (N)	Case (N)	Outcome	Balanced accuracy of class prediction			
				PLSDA	Lasso	RF	Combined
First parity							
T1-Precalving	64	50	New SH	0.52	0.51	0.51	0.51
		11	New SU	0.50	0.50	0.50	0.50
T2-Calving	64	50	New SH	0.55	0.55	0.50	0.53
		11	New SU	0.56	0.57	0.50	0.54
T3-Early	60	47	New SH	0.57	0.53	0.47	0.52
		11	New SU	0.60	0.57	0.50	0.56
≥Third parity							
T2-Calving	49	25	New SH	0.63	0.54	0.58	0.58
		12	New SU	0.71	0.54	0.53	0.59
T3-Early	48	26	New SH	0.57	0.54	0.50	0.54
		10	New SU	0.71	0.55	0.53	0.60
All parities							
T2-Calving	113	75	New SH	0.56	0.53	0.56	0.55
		23	New SU	0.53	0.53	0.50	0.52
		98	New SH/SU	0.57	0.54	0.57	0.56
T3-Early	108	73	New SH	0.56	0.56	0.50	0.54
		21	New SU	0.64	0.63	0.50	0.59
		94	New SH/SU	0.60	0.58	0.52	0.57
		35	All SU	0.66	0.65	0.54	0.62

1 Data were analyzed in 17 prespecified subsets split by time point, parity, and outcome definition; the control group in all cases were animals without sole lesions, and outcomes were cases of new sole hemorrhage (New SH), cases of new sole ulcers (New SU), those 2 groups combined (New SH/SU), or all cases of sole ulcers (All SU).

The stability of variable selection in bootstrapped resamples was calculated for each variable in each of the 17 subsets, resulting in 1,445 observed stabilities (Supplemental Table S4, https://data.mendeley.com/datasets/dgrb5z38kb/1; Barden, 2023). Baseline stability thresholds were calculated in each subset after permuting the outcome; T_99_ and T_100_ thresholds ranged from 73.7 to 91.8% and from 81.8 to 94.3%, respectively (Supplemental Table S4).

**Figure 4:**
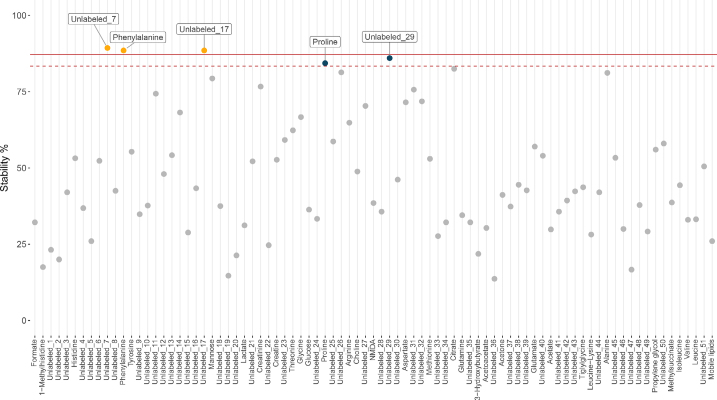
Variable selection stability for all cases of sole ulcers compared with unaffected cows in all parities in early lactation (T3-Early). The solid line is the T_100_ threshold, and the dashed line is the T_99_ threshold. T_99_ and T_100_ are the baseline stability thresholds equivalent to expected 1% and 0% false-positive rates, respectively.

**Figure 5:**
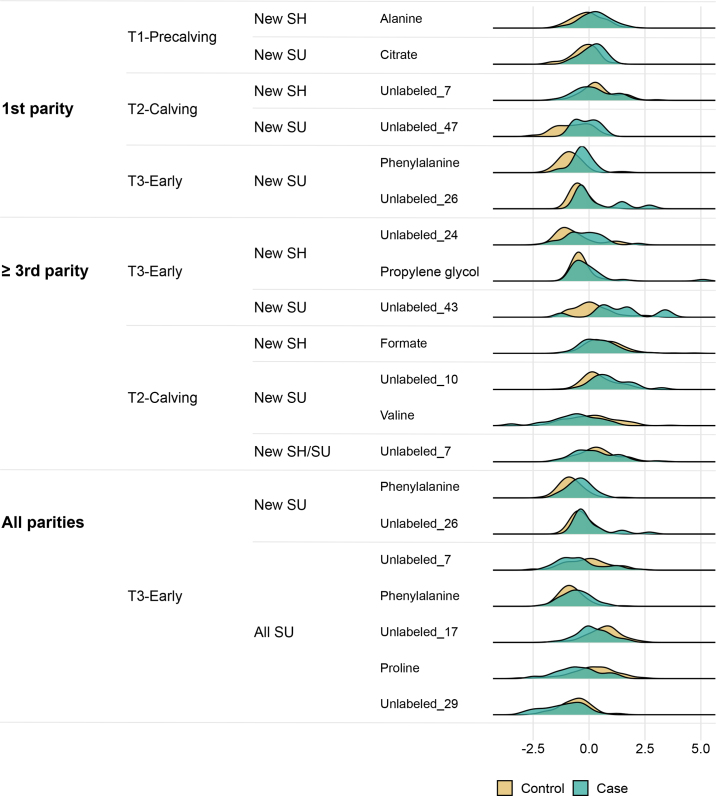
Density plots of standardized and mean-centered relative intensities of the metabolites in Table 5. SH = sole hemorrhage; SU = sole ulcer.

**Figure 6:**
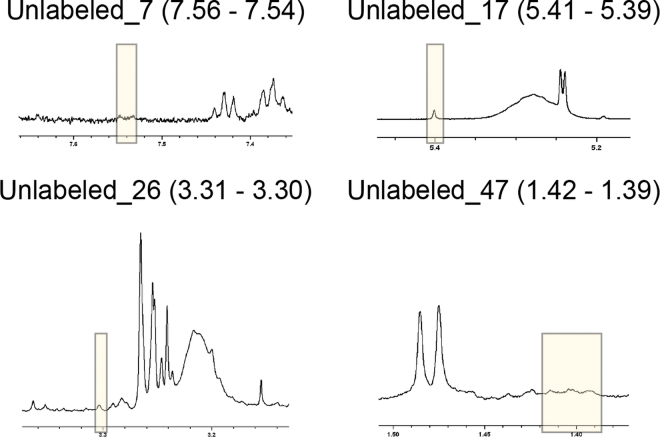
Example spectra and bin boundaries (yellow shading) for the 4 unlabeled metabolites with an observed stability greater than the T_100_ threshold (Table 5). T_100_ is the baseline stability threshold equivalent to an expected 0% false-positive rate.

As an example, Figure 4 shows the distribution of observed stabilities from the subset relating to all parities at T3-Early, comparing all cases of SU (new SU and chronic SU) to unaffected cows. Across all subsets (i.e., 1,445 observed stabilities), 20 variables had an observed stability above the corresponding T_99_ threshold for that subset, representing 15 different metabolites (Table 5). The distribution of the relative intensities of these metabolites in case and control samples are displayed in Figure 5. Only 9 variables had an observed stability greater than the corresponding T_100_ threshold in that subset, representing phenylalanine and 4 unlabeled metabolites. Figure 6 displays the spectra and bin boundaries of the 4 unlabeled metabolites represented by variables with an observed stability greater than the T_100_ threshold.

**Table 5:** Variable selection stability of variables that had a stability greater than the T_99_ threshold^1^

Time point	Outcome	Metabolite	Log_2_FC	Stability (%)	T_99_ (%)	T_100_ (%)
First parity						
T1-Precalving	New SH	Alanine	0.07	88.2	84.8	89.2
	New SU	Citrate	0.14	77.5	75.8	82.8
T2-Calving	New SH	Unlabeled_7*	−0.15	93.2	87.3	91.5
	New SU	Unlabeled_47*	0.09	83.7	73.7	81.8
T3-Early	New SU	Phenylalanine	0.20	81.2	75.6	83.7
		Unlabeled_26*	0.35	92.5		
≥Third parity						
T3-Early	New SH	Unlabeled_24	0.09	78.3	77.5	85.3
		Propylene glycol	0.17	80.5		
	New SU	Unlabeled_43	0.13	75.3	74.6	82.3
All parities						
T2-Calving	New SH	Formate	−0.14	90.8	89.4	91.3
	New SU	Unlabeled_10	0.12	80.8	78.8	82.7
		Valine	−0.13	82.0		
	New SH/SU	Unlabeled_7*	−0.08	93.7	91.8	93.5
T3-Early	New SU	Phenylalanine*	0.16	94.2	81.8	84.0
		Unlabeled_26*	0.18	86.3		
	All SU	Unlabeled_7*	−0.11	89.3	83.4	87.2
		Phenylalanine*	0.08	88.5		
		Unlabeled_17*	−0.17	88.5		
		Proline	−0.13	84.3		
		Unlabeled_29	−0.08	86.0		

1 Details include the mean log (base 2) fold-change (Log_2_FC) and the baseline stability thresholds (T_99_ and T_100_, equivalent to expected 1% and 0% false-positive rates, respectively). Data were analyzed in 17 prespecified subsets split by time point, parity, and outcome definition; the control group in all cases were animals without sole lesions, and outcomes were either cases of sole new hemorrhage (New SH), cases of new sole ulcers (New SU), those 2 groups combined (New SH/SU), or all cases of sole ulcers (All SU).

* Variables with stability greater than the T_100_.

## DISCUSSION

We designed a study to explore the association between the serum metabolome and the development of sole lesions in Holstein cows. Our results indicated that the serum metabolome, as characterized by ^1^H NMR spectroscopy, could not reliably discriminate between animals based on the presence or future development of sole lesions. Additionally, we identified only a small number of metabolites that may be associated with sole lesion development. Taken together, there is limited support from this study for a major metabolic component in the etiopathogenesis of sole lesions; however, we draw this conclusion cautiously because there are several important caveats to these results.

### Class Prediction

The average balanced accuracy of predictive modeling did not exceed 62% in any subset, although the balanced accuracy of PLSDA reached 71% on 2 occasions. Furthermore, as the predictive performance was assessed from cross-validation during the tuning of model parameters, estimates of the balanced accuracy may be upwardly biased (Varma and Simon, 2006). Balanced accuracy was highest in subsets where the outcome was concurrent SU, compared with time points before lesion development or when the outcome was SH rather than SU; however, our results suggest a limited potential to predict sole lesions from the serum metabolome.

The poor balanced accuracy of class prediction we observed is in contrast to results reported from 2 studies that analyzed the serum metabolome of lame cows using MS-based techniques (Dervishi et al., 2020; Zhang et al., 2020b), but, as the cause of lameness in these cows was not described, these results might not be comparable to ours. The metabolites that were most influential in these predictive models were lysine, leucine, and isoleucine (Zhang et al., 2020b) and valine, mannose, and phosphoric acid (Dervishi et al., 2020). Except for phosphoric acid, these metabolites were represented by explanatory variables in our data set but not highlighted as informative in our analyses. It would be useful to apply MS-based metabolomics toward lesion-specific causes of lameness in dairy cattle in future studies.

We highlighted potential technical causes of extraneous spectral variation; however, PCA did not indicate these to be influential in this regard. Teahan et al., 2006, Tibshirani, 1996 examined the effect of several experimental factors on NMR spectra and found interindividual variation to be the most influential. Compared with other farmed species and humans, dairy cattle have exceptionally high metabolic demands in early lactation (Webster, 2020), and individual-specific responses in this period could present a challenging degree of interindividual variation in metabolomic studies of early lactation dairy cows (Ghaffari et al., 2019; Luke et al., 2020). No strong differences in body condition or milk yield were observed between cases and controls, and these factors also did not appear influential with PCA. However, the serum metabolome is also affected by a range of clinical and subclinical metabolic diseases such as retained placentas and subclinical ketosis, which may occur parallel to sole lesion development in early lactation (Sun et al., 2014; Dervishi et al., 2017, 2018; Yong et al., 2021). As we were unable to monitor the study population closely enough to record all such conditions, it is unknown how their incidence may have affected our results.

Possible reasons for the poor prediction results in our study include the absence of any true association between the serum metabolome and sole lesions and insufficient study power. Power analysis of our data set with MetaboAnalyst (Pang et al., 2021) indicated that several hundred samples would be required in each group (depending on the subset) to achieve a study power of 0.8. Limited study power may be due to the small differences we observed between groups, which had large within-group variability (Figure 5). One strategy to increase study power would be to attempt to reduce the variability within each group and thus increase the relative magnitude of any effect. Therefore, future studies may benefit from applying stricter inclusion criteria and attempting to record all periparturient diseases in detail.

### Variable Selection

Across all subsets, only 9 variables had observed stability greater than the T_100_ threshold; these corresponded to 5 metabolites: phenylalanine and 4 unlabeled metabolites (Table 5). Selected variables should be interpreted in the context of the poor balanced accuracies of class prediction, which suggest that even if these variables are truly associated with sole lesions, they explain only a small part of the differences between affected and unaffected animals.

We used additional steps to minimize false-positive results from the variable selection. It is suggested that data analysis should integrate multiple methods (triangulation) that have different and unrelated sources of bias to avoid interpreting spurious results as informative (Munafò and Davey Smith, 2018). We took the average variable stability from multiple variable selection methods and compared this observed stability to the distribution of baseline stabilities in permuted data. This approach is reported to be robust to false-positive results from variable selection (Lima et al., 2021; Hyde et al., 2022; M. Green, unpublished data); however, as we used different approaches, such as PLSDA and Boruta, we cannot claim to have replicated this method exactly. Nevertheless, we would expect the principles of stability, triangulation, and permuted baseline stabilities to be generalizable across a range of analytic methods.

The T_99_ threshold was calculated from the 99th percentile in the distribution of baseline stabilities in each subset; therefore, we considered this threshold to translate to an expected false-positive rate of 1%. By analyzing 85 explanatory variables in 17 subsets, we calculated a total of 1,445 observed stabilities. If no variables had a relationship with the outcome, we would still expect around 1% of variables to have an observed stability greater than the T_99_ threshold. Across all subsets, 20 variables had an observed stability that exceeded the T_99_ threshold, which is, therefore, more than would be expected in the complete absence of any informative explanatory variables. However, many of these variables could still be false positives, so we focused our interpretation on the variables that had an observed stability above the T_100_ threshold, as these were the least likely to be artifactual. We have reported all variables with an observed stability greater than the T_99_ threshold so that future studies can determine whether any of these more equivocal results are verifiable.

Phenylalanine was highlighted as informative in subsets related to concurrent SU and had a higher concentration in animals with SU compared with unaffected cows; Dervishi et al., (2020) observed the same trend between lame and nonlame cows. Phenylalanine has been reported to be increased in humans due to inflammation and oxidative stress associated with conditions such as trauma, sepsis, and burns (Rath et al., 1987; Ploder et al., 2008). There is interest in the potential role of periparturient inflammation and oxidative stress on sole lesion development in dairy cattle (Al-Qudah and Ismail, 2012; Watson et al., 2022; Wilson et al., 2022).

Unlabeled metabolites were spectral signals that could not be annotated from a mammalian metabolite library. Example spectra of the 4 unlabeled metabolites that had an observed stability greater than the T_100_ threshold are shown in Figure 6. Two of these unlabeled metabolites represent distinct single peaks (Unlabeled_17 and Unlabeled_26), whereas the other 2 (Unlabeled_7 and Unlabeled_47) are much more poorly defined. By overlaying the spectra from all 567 samples, it was possible to visualize small but consistent changes in the baseline. These weak signals could relate to metabolites at the lower limit of detection by ^1^H NMR spectroscopy, metabolites occluded by the presence of albumin in serum, or signals attenuated during the CPMG pulse program; either way, their interpretation requires caution due to the low signal-to-noise ratio. Such metabolites may be more reliably detected and identified using serum extracts (which separate metabolites from albumin), other NMR pulse sequences, 2-dimensional NMR, or MS-based techniques (Beckonert et al., 2007; Marchand et al., 2017; Emwas et al., 2019).

### Generalizability

We enrolled cows from a single herd and, as a consequence, the generalizability of our study is intrinsically limited. The herd in our study operated under a typical but relatively intensive management system of zero-grazing, and this is an important context from which to interpret our results. The housing environment is a major risk factor for sole lesions (Cook et al., 2004; Cook and Nordlund, 2009; Bergsten et al., 2015), and the etiology and frequency of lameness differ between grazed and housed herds (Hund et al., 2019).

The average herd prevalence of lame cows in the UK has been reported from cross-sectional studies to be approximately 30% (Griffiths et al., 2018; Randall et al., 2019); in our study, we recorded the prevalence of lame cows to be between 7.5 and 10.7% depending on the time point of the study (data not shown). Previous studies in the UK that recorded foot lesions in first-parity animals observed that more than 95% of heifers were affected with sole lesions in early lactation (Leach et al., 1997; Maxwell et al., 2015; Randall et al., 2016); 63% of heifers were affected with sole lesions at the T3-Early assessment in our study. Taken together, we conclude that our study population was from a herd with better overall management of lameness than many UK dairy herds.

## CONCLUSIONS

We compared the serum metabolome in dairy cows that developed sole lesions in early lactation with that of unaffected cows. Analysis of the serum metabolome could not reliably predict the presence or future development of sole lesions. We also only highlighted a small number of metabolites that may be associated with sole lesion development. We conclude that the serum metabolome, as characterized by ^1^H NMR spectroscopy, is not strongly associated with sole lesion development, but any true association may have been masked by unrecorded variation between individual animals or from other experimental sources. The application of metabolomics undoubtedly has the potential to reveal underlying mechanisms of sole lesion etiopathogenesis in dairy cows; however, our results were equivocal in this respect and further studies would be beneficial.
